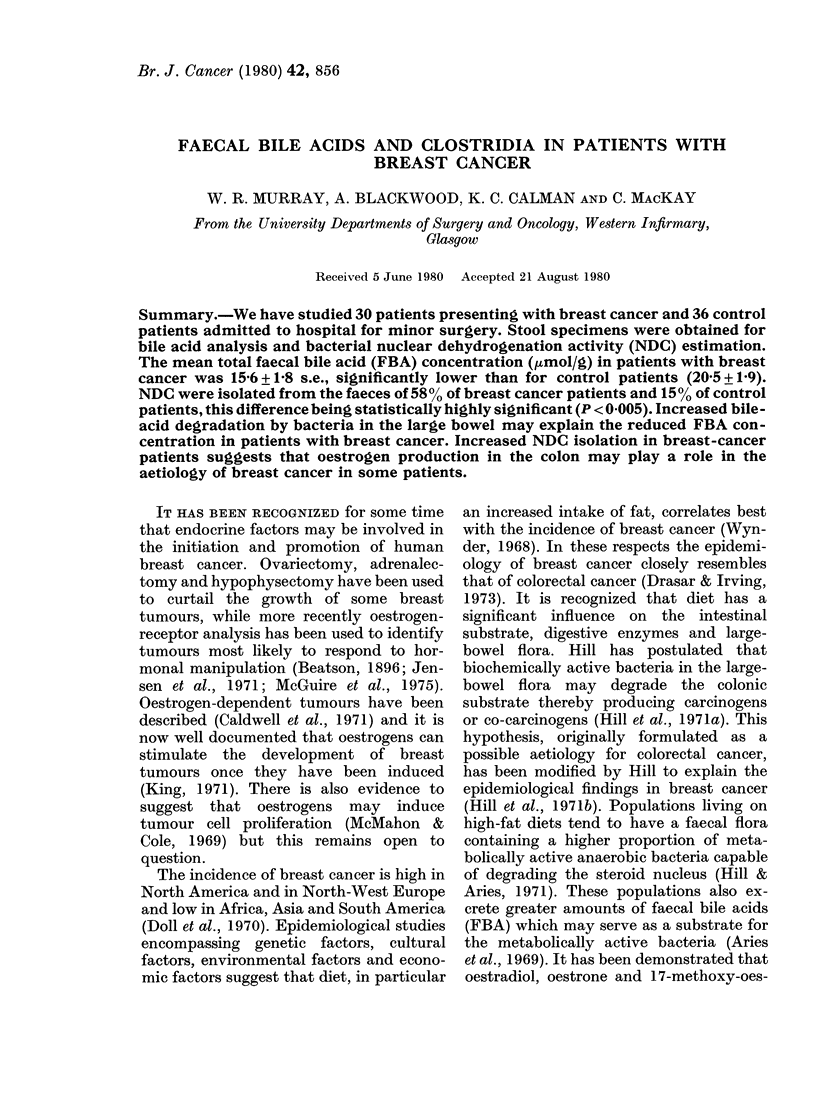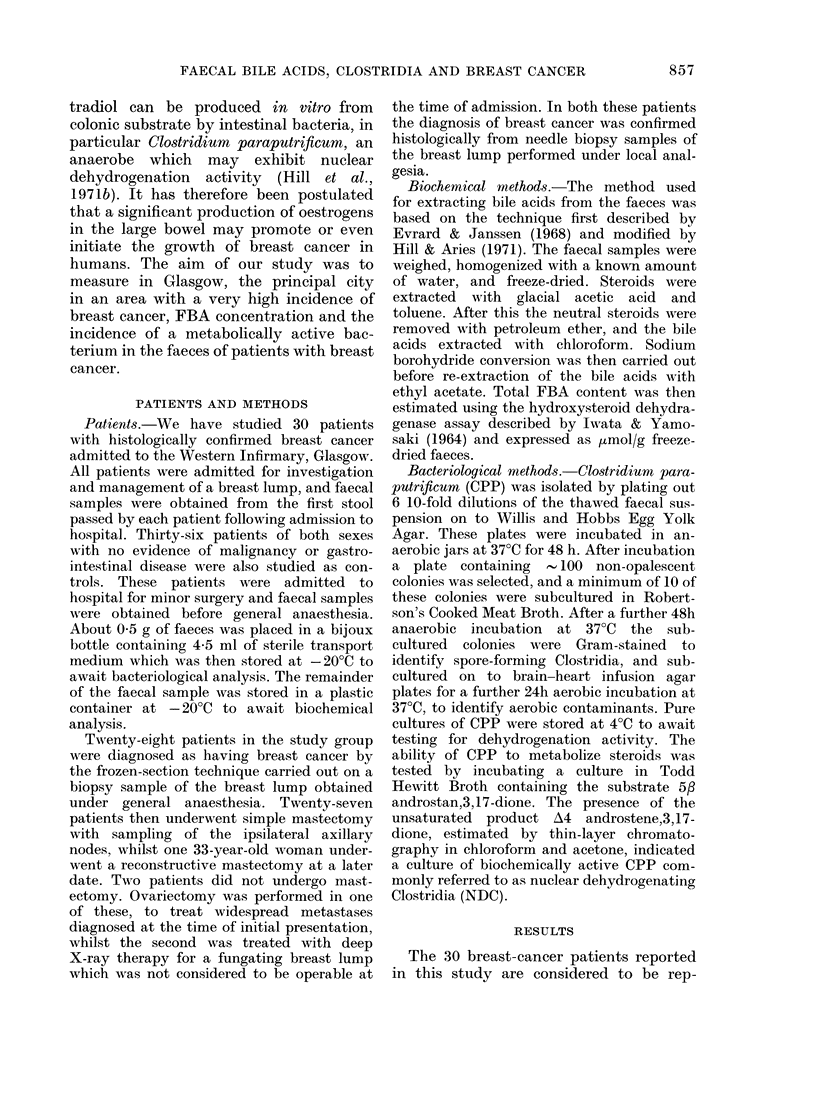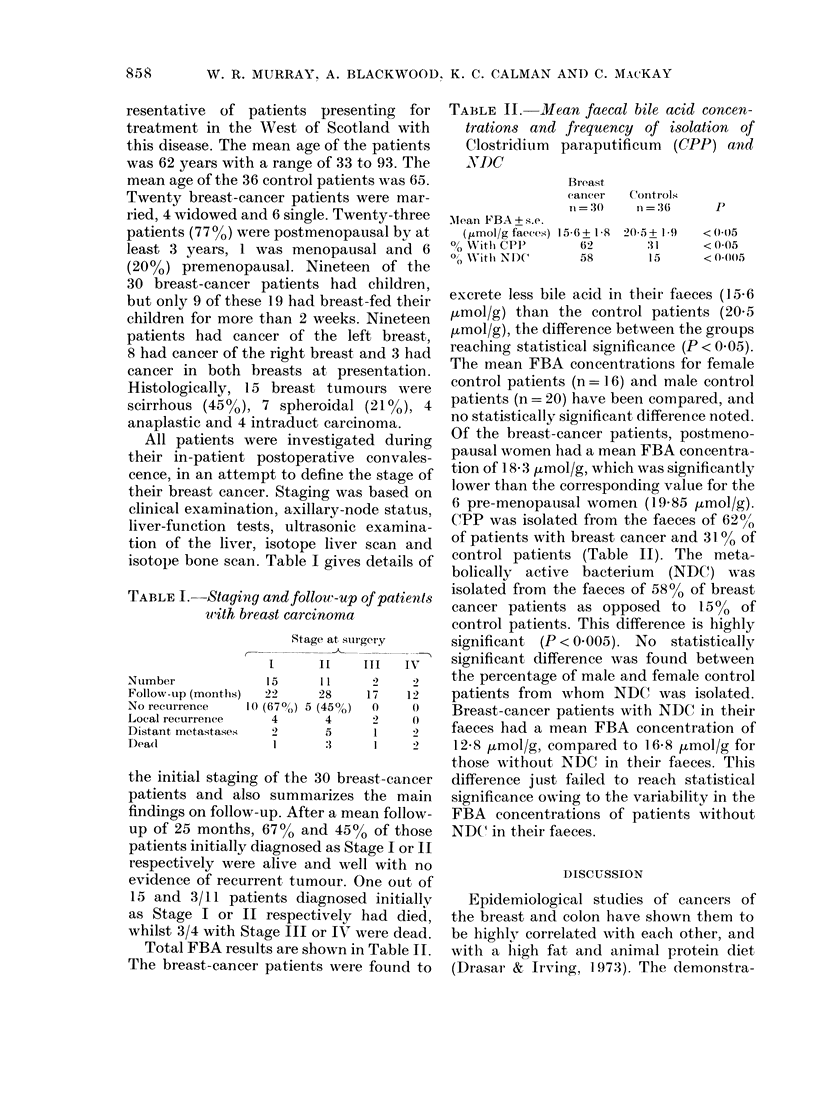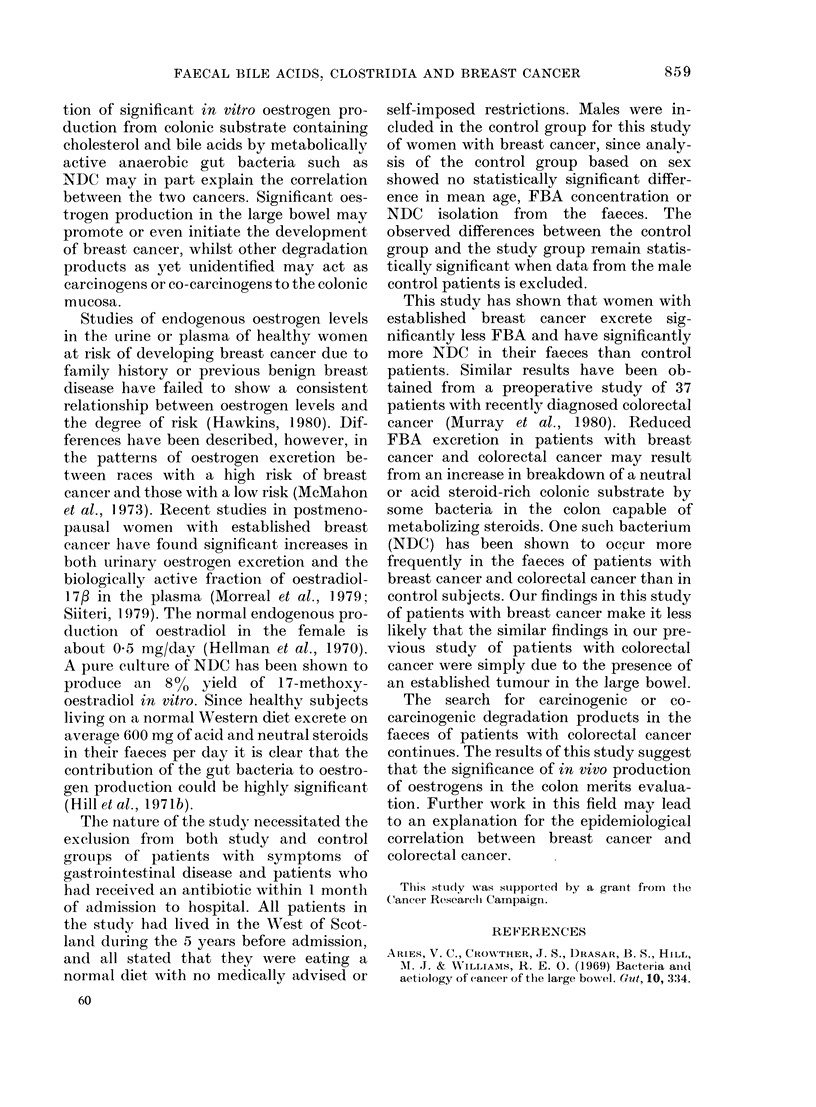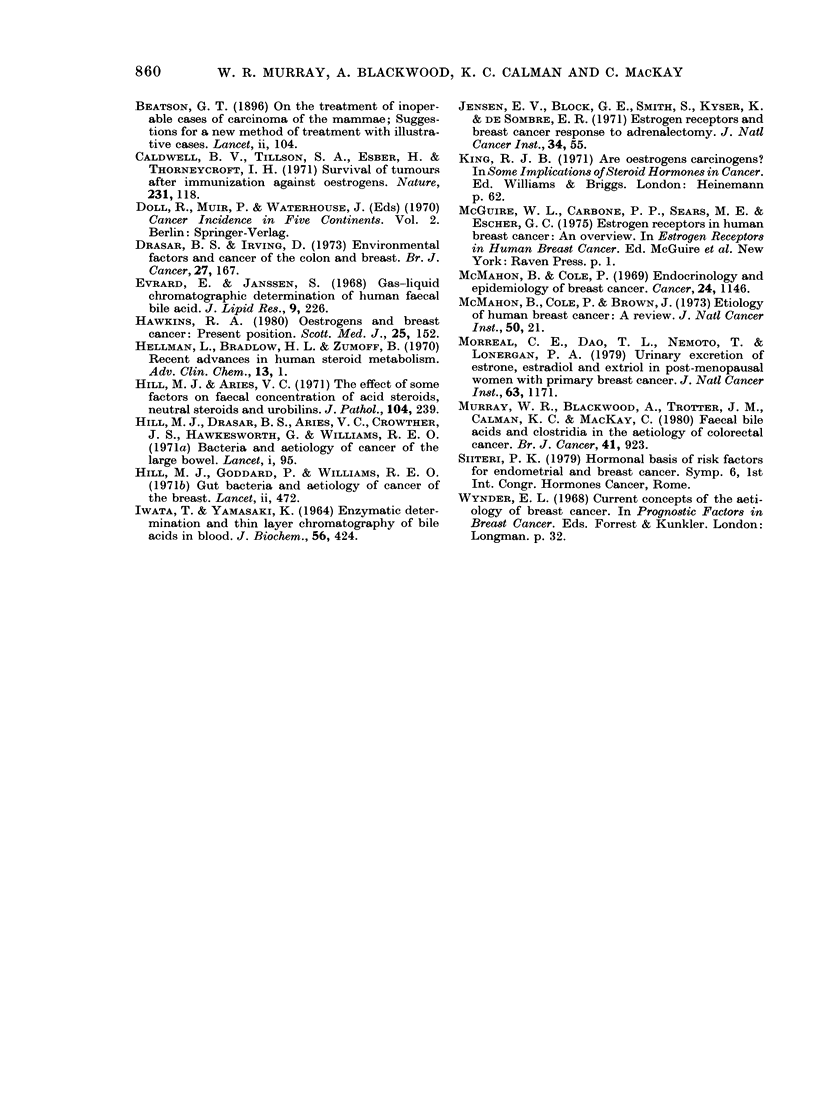# Faecal bile acids and clostridia in patients with breast cancer.

**DOI:** 10.1038/bjc.1980.333

**Published:** 1980-12

**Authors:** W. R. Murray, A. Blackwood, K. C. Calman, C. MacKay

## Abstract

We have studied 30 patients presenting with breast cancer and 36 control patients admitted to hospital for minor surgery. Stool specimens were obtained for bile acid analysis and bacterial nuclear dehydrogenation activity (NDC) estimation. The mean total faecal bile acid (FBA) concentration (mumol/g) in patients with breast cancer was 15.6 /+- 1.8 s.e., significantly lower than for control patients (20.5 /+- 1.9). NDC were isolated from the faeces of 58% of breast cancer patients an 15% of control patients, this difference being statistically highly significant (P less than 0.005). Increased bile-acid degradation by bacteria in the large bowel may explain the reduced FBA concentration in patients with breast cancer. Increased NDC isolation in breast-cancer patients suggests that oestrogen production in the colon may play a role in the aetiogy of breast cancer in some patients.


*Br. J. Cancer* (1980) **42**, 856

### FAECAL BILE ACIDS AND CLOSTRIDIA IN PATIENTS WITH BREAST CANCER

W. R. MURRAY, A. BLACKWOOD, K. C. CALMAN AND C. MACKAY

*From the University Departments of Surgery and Oncology, Western Infirmary, Glasgow*

Received 5 June 1980 Accepted 21 August 1980

**Summary.—We have studied 30 patients presenting with breast cancer and 36 control patients admitted to hospital for minor surgery. Stool specimens were obtained for bile acid analysis and bacterial nuclear dehydrogenation activity (NDC) estimation. The mean total faecal bile acid (FBA) concentration (μmol/g) in patients with breast cancer was 15·6 ± 1·8 s.e., significantly lower than for control patients (20·5 ± 1·9). NDC were isolated from the faeces of 58% of breast cancer patients and 15% of control patients, this difference being statistically highly significant ($P < 0.005$). Increased bile-acid degradation by bacteria in the large bowel may explain the reduced FBA concentration in patients with breast cancer. Increased NDC isolation in breast-cancer patients suggests that oestrogen production in the colon may play a role in the aetiology of breast cancer in some patients.**

IT HAS BEEN RECOGNIZED for some time that endocrine factors may be involved in the initiation and promotion of human breast cancer. Ovariectomy, adrenalectomy and hypophysectomy have been used to curtail the growth of some breast tumours, while more recently oestrogen-receptor analysis has been used to identify tumours most likely to respond to hormonal manipulation (Beatson, 1896; Jensen *et al.*, 1971; McGuire *et al.*, 1975). Oestrogen-dependent tumours have been described (Caldwell *et al.*, 1971) and it is now well documented that oestrogens can stimulate the development of breast tumours once they have been induced (King, 1971). There is also evidence to suggest that oestrogens may induce tumour cell proliferation (McMahon & Cole, 1969) but this remains open to question.

The incidence of breast cancer is high in North America and in North-West Europe and low in Africa, Asia and South America (Doll *et al.*, 1970). Epidemiological studies encompassing genetic factors, cultural factors, environmental factors and economic factors suggest that diet, in particular an increased intake of fat, correlates best with the incidence of breast cancer (Wynder, 1968). In these respects the epidemiology of breast cancer closely resembles that of colorectal cancer (Drasar & Irving, 1973). It is recognized that diet has a significant influence on the intestinal substrate, digestive enzymes and large-bowel flora. Hill has postulated that biochemically active bacteria in the large-bowel flora may degrade the colonic substrate thereby producing carcinogens or co-carcinogens (Hill *et al.*, 1971*a*). This hypothesis, originally formulated as a possible aetiology for colorectal cancer, has been modified by Hill to explain the epidemiological findings in breast cancer (Hill *et al.*, 1971*b*). Populations living on high-fat diets tend to have a faecal flora containing a higher proportion of metabolically active anaerobic bacteria capable of degrading the steroid nucleus (Hill & Aries, 1971). These populations also excrete greater amounts of faecal bile acids (FBA) which may serve as a substrate for the metabolically active bacteria (Aries *et al.*, 1969). It has been demonstrated that oestradiol, oestrone and 17-methoxy-oestradiol can be produced *in vitro* from colonic substrate by intestinal bacteria, in particular *Clostridium paraputrificum*, an anaerobe which may exhibit nuclear dehydrogenation activity (Hill *et al.*, 1971*b*). It has therefore been postulated that a significant production of oestrogens in the large bowel may promote or even initiate the growth of breast cancer in humans. The aim of our study was to measure in Glasgow, the principal city in an area with a very high incidence of breast cancer, FBA concentration and the incidence of a metabolically active bacterium in the faeces of patients with breast cancer.

### PATIENTS AND METHODS

*Patients.*—We have studied 30 patients with histologically confirmed breast cancer admitted to the Western Infirmary, Glasgow. All patients were admitted for investigation and management of a breast lump, and faecal samples were obtained from the first stool passed by each patient following admission to hospital. Thirty-six patients of both sexes with no evidence of malignancy or gastro-intestinal disease were also studied as controls. These patients were admitted to hospital for minor surgery and faecal samples were obtained before general anaesthesia. About 0·5 g of faeces was placed in a bijoux bottle containing 4·5 ml of sterile transport medium which was then stored at $-20°C$ to await bacteriological analysis. The remainder of the faecal sample was stored in a plastic container at $-20°C$ to await biochemical analysis.

Twenty-eight patients in the study group were diagnosed as having breast cancer by the frozen-section technique carried out on a biopsy sample of the breast lump obtained under general anaesthesia. Twenty-seven patients then underwent simple mastectomy with sampling of the ipsilateral axillary nodes, whilst one 33-year-old woman underwent a reconstructive mastectomy at a later date. Two patients did not undergo mastectomy. Ovariectomy was performed in one of these, to treat widespread metastases diagnosed at the time of initial presentation, whilst the second was treated with deep X-ray therapy for a fungating breast lump which was not considered to be operable at the time of admission. In both these patients the diagnosis of breast cancer was confirmed histologically from needle biopsy samples of the breast lump performed under local analgesia.

*Biochemical methods.*—The method used for extracting bile acids from the faeces was based on the technique first described by Evrard & Janssen (1968) and modified by Hill & Aries (1971). The faecal samples were weighed, homogenized with a known amount of water, and freeze-dried. Steroids were extracted with glacial acetic acid and toluene. After this the neutral steroids were removed with petroleum ether, and the bile acids extracted with chloroform. Sodium borohydride conversion was then carried out before re-extraction of the bile acids with ethyl acetate. Total FBA content was then estimated using the hydroxysteroid dehydragenase assay described by Iwata & Yamosaki (1964) and expressed as $\mu$mol/g freeze-dried faeces.

*Bacteriological methods.*—*Clostridium paraputrificum* (CPP) was isolated by plating out 6 10-fold dilutions of the thawed faecal suspension on to Willis and Hobbs Egg Yolk Agar. These plates were incubated in anaerobic jars at 37°C for 48 h. After incubation a plate containing $\sim$100 non-opalescent colonies was selected, and a minimum of 10 of these colonies were subcultured in Robertson's Cooked Meat Broth. After a further 48h anaerobic incubation at 37°C the subcultured colonies were Gram-stained to identify spore-forming Clostridia, and subcultured on to brain–heart infusion agar plates for a further 24h aerobic incubation at 37°C, to identify aerobic contaminants. Pure cultures of CPP were stored at 4°C to await testing for dehydrogenation activity. The ability of CPP to metabolize steroids was tested by incubating a culture in Todd Hewitt Broth containing the substrate 5$\beta$ androstan,3,17-dione. The presence of the unsaturated product $\Delta$4 androstene,3,17-dione, estimated by thin-layer chromatography in chloroform and acetone, indicated a culture of biochemically active CPP commonly referred to as nuclear dehydrogenating Clostridia (NDC).

### RESULTS

The 30 breast-cancer patients reported in this study are considered to be representative of patients presenting for treatment in the West of Scotland with this disease. The mean age of the patients was 62 years with a range of 33 to 93. The mean age of the 36 control patients was 65. Twenty breast-cancer patients were married, 4 widowed and 6 single. Twenty-three patients (77%) were postmenopausal by at least 3 years, 1 was menopausal and 6 (20%) premenopausal. Nineteen of the 30 breast-cancer patients had children, but only 9 of these 19 had breast-fed their children for more than 2 weeks. Nineteen patients had cancer of the left breast, 8 had cancer of the right breast and 3 had cancer in both breasts at presentation. Histologically, 15 breast tumours were scirrhous (45%), 7 spheroidal (21%), 4 anaplastic and 4 intraduct carcinoma.

All patients were investigated during their in-patient postoperative convalescence, in an attempt to define the stage of their breast cancer. Staging was based on clinical examination, axillary-node status, liver-function tests, ultrasonic examination of the liver, isotope liver scan and isotope bone scan. Table I gives details of

TABLE I.—*Staging and follow-up of patients with breast carcinoma*

| | Stage at surgery | | | |
|---|---|---|---|---|
| | I | II | III | IV |
| Number | 15 | 11 | 2 | 2 |
| Follow-up (months) | 22 | 28 | 17 | 12 |
| No recurrence | 10 (67%) | 5 (45%) | 0 | 0 |
| Local recurrence | 4 | 4 | 2 | 0 |
| Distant metastases | 2 | 5 | 1 | 2 |
| Dead | 1 | 3 | 1 | 2 |

the initial staging of the 30 breast-cancer patients and also summarizes the main findings on follow-up. After a mean follow-up of 25 months, 67% and 45% of those patients initially diagnosed as Stage I or II respectively were alive and well with no evidence of recurrent tumour. One out of 15 and 3/11 patients diagnosed initially as Stage I or II respectively had died, whilst 3/4 with Stage III or IV were dead.

Total FBA results are shown in Table II. The breast-cancer patients were found to

TABLE II.—*Mean faecal bile acid concentrations and frequency of isolation of* Clostridium paraputificum *(CPP) and NDC*

| | Breast cancer n = 30 | Controls n = 36 | *P* |
|---|---|---|---|
| Mean FBA ± s.e. (μmol/g faeces) | 15·6 ± 1·8 | 20·5 ± 1·9 | < 0·05 |
| % With CPP | 62 | 31 | < 0·05 |
| % With NDC | 58 | 15 | < 0·005 |

excrete less bile acid in their faeces (15·6 μmol/g) than the control patients (20·5 μmol/g), the difference between the groups reaching statistical significance ($P < 0·05$). The mean FBA concentrations for female control patients (n = 16) and male control patients (n = 20) have been compared, and no statistically significant difference noted. Of the breast-cancer patients, postmenopausal women had a mean FBA concentration of 18·3 μmol/g, which was significantly lower than the corresponding value for the 6 pre-menopausal women (19·85 μmol/g). CPP was isolated from the faeces of 62% of patients with breast cancer and 31% of control patients (Table II). The metabolically active bacterium (NDC) was isolated from the faeces of 58% of breast cancer patients as opposed to 15% of control patients. This difference is highly significant ($P < 0·005$). No statistically significant difference was found between the percentage of male and female control patients from whom NDC was isolated. Breast-cancer patients with NDC in their faeces had a mean FBA concentration of 12·8 μmol/g, compared to 16·8 μmol/g for those without NDC in their faeces. This difference just failed to reach statistical significance owing to the variability in the FBA concentrations of patients without NDC in their faeces.

### DISCUSSION

Epidemiological studies of cancers of the breast and colon have shown them to be highly correlated with each other, and with a high fat and animal protein diet (Drasar & Irving, 1973). The demonstration of significant *in vitro* oestrogen production from colonic substrate containing cholesterol and bile acids by metabolically active anaerobic gut bacteria such as NDC may in part explain the correlation between the two cancers. Significant oestrogen production in the large bowel may promote or even initiate the development of breast cancer, whilst other degradation products as yet unidentified may act as carcinogens or co-carcinogens to the colonic mucosa.

Studies of endogenous oestrogen levels in the urine or plasma of healthy women at risk of developing breast cancer due to family history or previous benign breast disease have failed to show a consistent relationship between oestrogen levels and the degree of risk (Hawkins, 1980). Differences have been described, however, in the patterns of oestrogen excretion between races with a high risk of breast cancer and those with a low risk (McMahon *et al.*, 1973). Recent studies in postmenopausal women with established breast cancer have found significant increases in both urinary oestrogen excretion and the biologically active fraction of oestradiol-17$\beta$ in the plasma (Morreal *et al.*, 1979; Siiteri, 1979). The normal endogenous production of oestradiol in the female is about 0·5 mg/day (Hellman *et al.*, 1970). A pure culture of NDC has been shown to produce an 8% yield of 17-methoxy-oestradiol *in vitro*. Since healthy subjects living on a normal Western diet excrete on average 600 mg of acid and neutral steroids in their faeces per day it is clear that the contribution of the gut bacteria to oestrogen production could be highly significant (Hill *et al.*, 1971*b*).

The nature of the study necessitated the exclusion from both study and control groups of patients with symptoms of gastrointestinal disease and patients who had received an antibiotic within 1 month of admission to hospital. All patients in the study had lived in the West of Scotland during the 5 years before admission, and all stated that they were eating a normal diet with no medically advised or self-imposed restrictions. Males were included in the control group for this study of women with breast cancer, since analysis of the control group based on sex showed no statistically significant difference in mean age, FBA concentration or NDC isolation from the faeces. The observed differences between the control group and the study group remain statistically significant when data from the male control patients is excluded.

This study has shown that women with established breast cancer excrete significantly less FBA and have significantly more NDC in their faeces than control patients. Similar results have been obtained from a preoperative study of 37 patients with recently diagnosed colorectal cancer (Murray *et al.*, 1980). Reduced FBA excretion in patients with breast cancer and colorectal cancer may result from an increase in breakdown of a neutral or acid steroid-rich colonic substrate by some bacteria in the colon capable of metabolizing steroids. One such bacterium (NDC) has been shown to occur more frequently in the faeces of patients with breast cancer and colorectal cancer than in control subjects. Our findings in this study of patients with breast cancer make it less likely that the similar findings in our previous study of patients with colorectal cancer were simply due to the presence of an established tumour in the large bowel.

The search for carcinogenic or co-carcinogenic degradation products in the faeces of patients with colorectal cancer continues. The results of this study suggest that the significance of *in vivo* production of oestrogens in the colon merits evaluation. Further work in this field may lead to an explanation for the epidemiological correlation between breast cancer and colorectal cancer.

This study was supported by a grant from the Cancer Research Campaign.

### REFERENCES

ARIES, V. C., CROWTHER, J. S., DRASAR, B. S., HILL, M. J. & WILLIAMS, R. E. O. (1969) Bacteria and aetiology of cancer of the large bowel. *Gut*, **10**, 334.

BEATSON, G. T. (1896) On the treatment of inoperable cases of carcinoma of the mammae; Suggestions for a new method of treatment with illustrative cases. *Lancet*, ii, 104.

CALDWELL, B. V., TILLSON, S. A., ESBER, H. & THORNEYCROFT, I. H. (1971) Survival of tumours after immunization against oestrogens. *Nature*, **231**, 118.

DOLL, R., MUIR, P. & WATERHOUSE, J. (Eds) (1970) *Cancer Incidence in Five Continents*. Vol. 2. Berlin: Springer-Verlag.

DRASAR, B. S. & IRVING, D. (1973) Environmental factors and cancer of the colon and breast. *Br. J. Cancer*, **27**, 167.

EVRARD, E. & JANSSEN, S. (1968) Gas-liquid chromatographic determination of human faecal bile acid. *J. Lipid Res.*, **9**, 226.

HAWKINS, R. A. (1980) Oestrogens and breast cancer: Present position. *Scott. Med. J.*, **25**, 152.

HELLMAN, L., BRADLOW, H. L. & ZUMOFF, B. (1970) Recent advances in human steroid metabolism. *Adv. Clin. Chem.*, **13**, 1.

HILL, M. J. & ARIES, V. C. (1971) The effect of some factors on faecal concentration of acid steroids, neutral steroids and urobilins. *J. Pathol.*, **104**, 239.

HILL, M. J., DRASAR, B. S., ARIES, V. C., CROWTHER, J. S., HAWKESWORTH, G. & WILLIAMS, R. E. O. (1971*a*) Bacteria and aetiology of cancer of the large bowel. *Lancet*, i, 95.

HILL, M. J., GODDARD, P. & WILLIAMS, R. E. O. (1971*b*) Gut bacteria and aetiology of cancer of the breast. *Lancet*, ii, 472.

IWATA, T. & YAMASAKI, K. (1964) Enzymatic determination and thin layer chromatography of bile acids in blood. *J. Biochem.*, **56**, 424.

JENSEN, E. V., BLOCK, G. E., SMITH, S., KYSER, K. & DE SOMBRE, E. R. (1971) Estrogen receptors and breast cancer response to adrenalectomy. *J. Natl Cancer Inst.*, **34**, 55.

KING, R. J. B. (1971) Are oestrogens carcinogens? In *Some Implications of Steroid Hormones in Cancer*. Ed. Williams & Briggs. London: Heinemann p. 62.

MCGUIRE, W. L., CARBONE, P. P., SEARS, M. E. & ESCHER, G. C. (1975) Estrogen receptors in human breast cancer: An overview. In *Estrogen Receptors in Human Breast Cancer*. Ed. McGuire *et al.* New York: Raven Press. p. 1.

MCMAHON, B. & COLE, P. (1969) Endocrinology and epidemiology of breast cancer. *Cancer*, **24**, 1146.

MCMAHON, B., COLE, P. & BROWN, J. (1973) Etiology of human breast cancer: A review. *J. Natl Cancer Inst.*, **50**, 21.

MORREAL, C. E., DAO, T. L., NEMOTO, T. & LONERGAN, P. A. (1979) Urinary excretion of estrone, estradiol and extriol in post-menopausal women with primary breast cancer. *J. Natl Cancer Inst.*, **63**, 1171.

MURRAY, W. R., BLACKWOOD, A., TROTTER, J. M., CALMAN, K. C. & MACKAY, C. (1980) Faecal bile acids and clostridia in the aetiology of colorectal cancer. *Br. J. Cancer*, **41**, 923.

SIITERI, P. K. (1979) Hormonal basis of risk factors for endometrial and breast cancer. Symp. 6, 1st Int. Congr. Hormones Cancer, Rome.

WYNDER, E. L. (1968) Current concepts of the aetiology of breast cancer. In *Prognostic Factors in Breast Cancer*. Eds. Forrest & Kunkler. London: Longman. p. 32.